# Supplementary material for: Sensitive detection of rare disease-associated cell subsets via representation learning
Source: Nat Commun. 2017 Apr 6;8:14825. doi: 10.1038/ncomms14825 (PMC5384229; doi:10.1038/ncomms14825)
Supplement: Supplementary Information — Supplementary figures, supplementary note, supplementary methods and supplementary references. [file ncomms14825-s1.pdf]

Supplementary Note 1

Machine learning glossary

Unsupervised learning: a group of machine learning algorithms that aim at discovering patterns in unlabeled data. Unsupervised learning algorithms commonly used in computational biology include clustering (e.g. for definition of cell populations) as well as principal component analysis (PCA) and t-Distributed Stochastic Neighbor Embedding (t-SNE) projections for data visualization. The outlier detection and autoencoder training algorithms that are compared with CellCnn in Figure 5 are also examples of unsupervised learning techniques.

Supervised learning: a group of machine learning algorithms that operate on labeled data. The training input data consists of pairs (observation, label) and the task is to infer a model that is able to correctly predict the corresponding labels of new observations. Examples of supervised learning algorithms used in our study are logistic regression, support vector machines and random forests for prediction of categorical labels (Figure 5c) and linear or Cox regression for prediction of continuous labels. CellCnn and Citrus are also supervised learning algorithms, where each observation is a group of single-cell measurements (e.g. a cytometry sample) and each label is the corresponding phenotype.

Overfitting: a common pitfall in supervised machine learning problems. Overfitting occurs when the inferred model can correctly predict the labels of the observations used for training but it fails to correctly predict the labels of new observations. It is a common problem in computational biology due to the limited number of training samples often available.

Generalization performance: the ability of a trained model to correctly predict labels of unseen observations, i.e. to avoid *overfitting*.

Multiple instance learning: a variation of supervised learning where the input data consists of groups of observations and each group of observations is associated with a label. Notably, the labels characterize the whole group and not individual observations. Two tasks are typically defined in terms of such data: (a) infer a model that correctly predicts the label of unseen groups of observations and (b) infer a model that correctly predicts the label of individual observations. CellCnn is designed to solve a multiple instance learning problem where observations correspond to single-cell abundance profiles and a group of observations corresponds to e.g. a mass cytometry sample associated with a label (e.g. healthy/diseased).

Features: transformation of the raw data observations, used as input for machine learning algorithms. Examples would be the presence/absence of specific sequence motifs in genomic sequencing data, or the abundances of different cell types in a mass cytometry sample. The latter type of features is used by the Citrus algorithm.

Representation: a set of features representing the data of interest.

Feature engineering: the task of *a priori* defining a problem-specific representation of the data based on prior knowledge. Choosing the right features is often an important step to successfully apply a machine learning algorithm.

Representation learning: the task of automating the feature engineering procedure. Useful features are not *a priori* defined but rather learned directly from the raw data. Representation learning is often performed by *artificial neural networks*. CellCnn is a representation learning algorithm, where the learned features often correspond to phenotype-associated cell subpopulations.

Artificial neural network: a machine learning model originally inspired by the information-processing paradigm of the brain. The basic computational unit is called a *neuron*, and neurons are organized in *layers*. Parameters such as the number of layers, the number of neurons in each layer and possible constraints on the connectivity patterns between neurons define the *neural network architecture*. The neural network architecture dictates how the input signal is (non-linearly) transformed at the different network layers and is finally propagated to the network output, where a prediction is made. Because of the non-linear operations performed at each layer, the layers closer to the network output tend to learn increasingly more complex and abstract representations of the input data.

Convolutional neural network: artificial neural network specifically designed to process the two-dimensional structure of images. It typically consists of one or more sets of convolutional and pooling layers. The convolutional layer comprises *filters* that evaluate the occurrence of specific patterns in image patches (convolutional unit) and the pooling layer computes summaries of these occurrences. CellCnn implements a convolutional neural network whose convolutional units are single cell abundance profiles.

Filter: a computational unit with a local connectivity pattern encountered in convolutional neural networks. A filter operates locally on predefined convolutional units of a data instance (i.e. small two-dimensional image regions in an image or single-cell profiles in an input group of cells, see Figure 1b) by computing a weighted sum of its input, followed by a nonlinear operation. Notably, a filter uses a shared vector of weights across all convolutional units, i.e. the filter evaluates a specific pattern in the input data. Multiple filters are used to detect different patterns.

Denoising autoencoder: an unsupervised representation learning model [24] that is trained to reconstruct the original input from a corrupted version of it, e.g. after addition of gaussian noise. The goal of this training procedure is to learn a representation that is robust to small perturbations of the input data and, therefore, captures essential features of the input data distribution.

Supplementary Methods

Data preprocessing for mass cytometry samples

Mass cytometry measurements were transformed using the inverse hyperbolic sine (arcsinh) function with a cofactor of 5. In the PBMC experiment, we combined data from the same donor that were analyzed in different mass cytometry runs introducing batch effects (systematic shifts in univariate marker distributions between samples). We used manually gated CD4+ T-cells as reference for correcting these batch effects in intracellular marker expression. Assuming that the univariate marker distribution of a specific cell type in the unstimulated condition is identical across different runs, a shift was applied to marker expression values so that unstimulated CD4+ T-cells from all runs have the same mean expression. The run-specific shift applied to unstimulated CD4+ T-cells was then applied to all cells measured in the same run. For the PBMC, NK cell, AML and ALL analyses, all univariate marker distributions were scaled on the basis of the training data to have mean equal to 0 and standard deviation equal to 1.

Data preprocessing for the HIV-cohort dataset

Data compensation and gating of live T-cells was performed as described in the original study [3]. Raw measurements were transformed using the inverse hyperbolic sine (arcsinh) function with a cofactor of 150. Markers used for gating were excluded from our analysis. The remaining markers were scaled on the basis of the training data to have mean equal to 0 and standard deviation equal to 1.

Creating multi-cell inputs

We provide two different modes for creating multi-cell inputs: random and biased towards outlier observations.

random: Multi-cell inputs are sampled uniformly at random from the original samples. We used this mode for the PBMC, HIV and NK cell datasets.

biased towards outliers: When expecting informative cell populations with very low frequency (< 0.1%), we used a biased procedure for creating multi-cell inputs from the non-control samples so as to enrich for presence of "abnormal" cells. 50% of a multi-cell input was sampled uniformly at random from the whole cell population whereas the other 50% was sampled from cells with high *outlierness score*. We used the top 10% outlier cells and sampled from them with probability proportional to their outlierness score. The algorithm used for outlier detection is described in (Supplementary Methods, subsection outlier detection).

CellCnn network architecture and hyperparameter selection

CellCnn is a convolutional neural network with one convolutional, one pooling and one output layer. The convolutional layer has a predefined number of filters and each filter computes a weighted sum of its input vector (single cell profile) followed by a nonlinear operation, namely $ReLU(x) = max(0, x)$. Pooling is performed separately for each convolutional filter, but across all cells of the multi-cell input. The pooling layer is connected to the output layer, which differs between classification and regression problems. For

classification, each class is represented by an output node with softmax nonlinearity and training is performed via minimizing the categorical cross entropy cost function. For regression, the output layer comprises one node with hyperbolic tangent nonlinearity and training is performed via minimizing the mean square error between the network prediction and the true label.

The objective that is minimized while training a neural network is highly non-convex and, thus, the network might easily underfit (get stuck in a local optimum) or overfit (perform very well on the training data but not be able to generalize to new data). A validation set is necessary to control this behaviour. Multi-cell inputs from the validation set are not used for training, but instead are used to monitor the predictive performance of the neural network after each training loop (epoch) and select the best-performing model.

In our experiments, we used random search to optimize a set of important hyperparameters (number of filters, learning rate, dropout), whereas the remaining ones were kept fixed (mini-batch size = 128, maximum number of training epochs = 100, early stopping after 5 epochs, initial weights sampled from a uniform distribution $U(-0.01, 0.01)$, L_2 weight decay = 1e-04). During random search, the learning rate was sampled on a logarithmic scale from the range [0.001, 0.1] and the number of filters was selected uniformly at random from a list of choices: (2, 3, 4, 5, 10, 20, 50). Dropout regularization was performed when including 10 filters or more to avoid spurious co-adaptation between filters [4].

Analysis of the PBMC dataset

Multi-cell inputs: Each sample was initially split into a training (80%) and a validation (20%) set of cells. Within the training/validation sets, 4096 multi-cell inputs, each comprising 200 cells were drawn for each phenotype.

Best-performing network: The convolutional layer comprised 2 filters. Max-pooling was performed on the output of each filter in order to detect *presence* of stimulated cells.

Analysis of the HIV-cohort dataset

From the initial cohort of 416 patients, we excluded those with negative disease-onset survival times and also those with samples containing fewer than 3000 cells. The resulting cohort comprised 383 patients, who were split into training (⅔, 256 individuals) and test set (⅓, 127 individuals). We used stratified cross validation to ensure that the same ratio of censored and uncensored events is present in the training and test set. Within the training set, only samples from patients with uncensored survival times were used for fitting the model, since the mean-squared-error objective used for training the neural network is not compatible with censored data. Uncensored survival times were ranked and mapped to the [-1, 1] interval and subsequently used as labels to be compared with network output.

Multi-cell inputs: 400 multi-cell inputs, each comprising 500 cells, were generated per patient.

Best-performing network: The best performing architecture comprised 3-5 filters (varying among cross validation runs). Mean-pooling was performed on the output of each filter in order to detect varying cell population *frequencies* across samples. We found that mean-pooling of the top 50 cells for each filter combined with regularization of the cell filter activity enables the network to identify a greater variety of cell types and therefore adopted this strategy.

Cross validation and representative filters: 5-fold cross validation (CV) was used on the training set in order to build a model that would be evaluated on the test set. Patients were initially split into 5 folds. The cross validation scheme was designed so that each fold contained multi-cell inputs from different patients. For each cross validation fold, we trained 10 networks with varying number of filters and learning rate and selected the one with best predictive performance on the validation set of the respective fold. The 5-fold cross validation procedure resulted in 5 different networks, which were further analyzed in order to select frequently occurring filters. We compiled a matrix of all filter weights from the 5 networks and performed hierarchical clustering using cosine similarity as metric. For each cluster of filters with at least 2 members, a cluster-specific cell population was defined as the intersection of the sets of cells selected by the filters belonging to that cluster. The reported phenotype-associated cell populations correspond to the cluster-specific cell populations defined using the above strategy.

Predictions on the test cohort: An ensemble model, consisting of the 5 best networks (one from each cross validation fold), was used to predict survival times for the individuals in the test cohort. For the test phase, one subset of 3000 cells was used per individual. The output of CellCnn corresponded to predicted disease-free survival time for each patient and was used to split the test cohort into low- and high-risk groups. The threshold used for defining the two risk groups was the median predicted survival time.

Analysis of the NK cell benchmark study

Multi-cell inputs: 200 multi-cell inputs, each comprising 3000 cells, were generated per individual.

Best-performing network: The best performing architecture comprised 3-5 filters (varying among Monte Carlo cross validation runs). Mean-pooling of the top 1% of cells was performed on the output of each filter.

Analysis of the AML rare-cell-type benchmark study

Multi-cell inputs: For each phenotype, 4096 multi-cell inputs were used for training and 1024 for validation. Each multi-cell input comprised 1000 cells.

Best-performing network: The convolutional layer comprised 20 filters and training was performed using dropout regularization with dropout probability of 0.5.

Analysis of the "personalized medicine" study

Multi-cell inputs: In this setting, only a single sample is available per phenotype (healthy/diseased). For each sample, 4096 multi-cell inputs were used for training and 1024 for validation. Each multi-cell input comprised 1000 cells.

Best-performing network: The convolutional layer comprised 20 filters and training was performed using dropout regularization with dropout probability of 0.5.

Outlier detection

We used a distance-based outlier detection approach based on the method described in [5]. A set S of s observations (single cell profiles) is sampled uniformly at random from the *inlier* class (i.e. the healthy control samples) and then used to evaluate the *outlierness* of single cell profiles in the test samples. The outlierness of an observation is defined as the L_1 distance between this observation and its closest neighbour in S. The size of the set S is the

only parameter of the algorithm that needs to be tuned. Our benchmark results (**Supplementary Fig. 6**) suggest that setting $s \geq 100{,}000$ achieves best performance on mass cytometry data. We have used $s = 200{,}000$ for the comparison with CellCnn.

Multi-cell input generation: When creating multi-cell inputs biased toward outlier observations, we used $s = 100$ and sampled the set S using the kmeans++ initialization strategy in order to get a more uniform coverage of the reference class. This choice achieved a good balance between sensitive outlier detection and low running time. As a preprocessing step for outlier detection, values lower than the 0.5^{th} percentile or higher than the 99.5^{th} percentile of each univariate marker distribution were normalized to the $0.5^{th}/99.5^{th}$ percentile values of the reference class, respectively. This preprocessing step prioritizes as outliers cells with abnormal combinations of markers, rather than cells with extreme values in e.g. one marker.

Parameter settings for Citrus

HIV-cohort study: We have performed the analysis described in the original Citrus publication [2] on the same random data partition - into training/test cohort - as the one used with CellCnn. The analysis was performed using Citrus version v0.7 because the latest version v0.8 does not support survival analysis with censored data. In summary, 3000 cells were drawn per sample and the minimum cluster size threshold was set to 0.5% of the total cell events. Cluster frequencies on a per sample basis were computed as features and fed into a L_1 penalized Cox regression model. 10-fold cross validation was used on the training set to determine the optimal penalty term for the regularized Cox model. Subsequently, 3000 cells were drawn from each test sample and mapped to the training clusters. Cluster frequency-based features were extracted from the test samples and were subsequently used as input to the regularized Cox regression model, in order to compute the predicted relative risk for each individual in the test cohort.

NK cell study: The analysis was performed using the latest Citrus version v0.8. The same 100 Monte Carlo cross validation partitions into training and test set, that were used for CellCnn, were also used for Citrus. During training, Citrus was instructed to pre-select 20,000 cells from each sample and used a minimum cluster size threshold of 0.05% of the total cell events. Cluster abundances on a per sample basis were computed as features and used as input to a L_1 penalized logistic regression model. 3-fold cross validation was used on the training set to determine the optimal penalty term for the regularized logistic regression model. For testing, 20,000 cells were drawn uniformly at random from each test sample and mapped to the training clusters. Cluster frequency-based features were extracted from the test samples and were subsequently used as input to the regularized logistic regression model, in order to compute the predicted CMV serology status for each individual in the test cohort.

AML rare cell type study: The analysis was performed using the latest Citrus version v0.8. Samples from the predefined training and validation sets (see **Methods**, section Datasets) were provided for training. For a three-class classification problem, Citrus uses the nearest shrunken centroid method and ideally needs at least 8 samples per class in order to avoid spurious results [2]. We provided 15 samples from each class (healthy, CN AML, CBF AML) by randomly splitting the original samples into non-overlapping parts. During training, Citrus was instructed to pre-select 10,000 cells from each sample and used a minimum cluster size threshold of 0.03% of the total cell events. Cluster abundances on a per sample basis were

computed as features and used as input for the nearest shrunken centroid algorithm. For testing, all cells from the test samples were mapped to the closest cluster defined during training and, subsequently, cluster abundances were computed on a per sample basis.

Parameter settings for baseline methods

Single-cell input logistic regression / SVM / random forests: a logistic regression / support vector machine / random forests classifier that takes as input single cell profiles from the multi-cell inputs generated for CellCnn. Each single cell profile is labeled with the label (e.g. disease condition, survival time) of its corresponding cytometry sample. Random search was used to optimize over the following hyperparameters of the classifiers: type (L_1/L_2) and strength of weight regularization for logistic regression and SVM, number of trees (20 trees finally used) and tree-specific parameters (such as maximum depth, minimum number of samples required to split an internal node, minimum number of samples in newly created leaves, function to measure the quality of a split) for random forests. We used the implementations of these algorithms available in the Python machine learning library scikit-learn.

Denoising autoencoder: an unsupervised representation learning model [6] that is trained to reconstruct the original input from a corrupted version of it, e.g. after addition of gaussian noise. We used the same multi-cell inputs and network architecture as for CellCnn, but removed the pooling layer and substituted the output layer by the multi-cell input. Random search was used to optimize over the number of filters (we considered 5, 10, 20, 50 or 100 filters) and the standard deviation of gaussian noise added to the input. The standard deviation was drawn from a uniform distribution $U(0.1, 0.5)$.

Supplementary References

1. Bodenmiller, B. et al. Multiplexed mass cytometry profiling of cellular states perturbed by small-molecule regulators. Nat. Biotechnol. 30, 858–867 (2012).

2. Bruggner, R. V., Bodenmiller, B., Dill, D. L., Tibshirani, R. J. & Nolan, G. P. Automated identification of stratifying signatures in cellular subpopulations. Proc. Natl. Acad. Sci. U. S. A. 111, E2770–7 (2014).

3. Weintrob, A. C. et al. Increasing age at HIV seroconversion from 18 to 40 years is associated with favorable virologic and immunologic responses to HAART. J. Acquir. Immune Defic. Syndr. 49, 40–47 (2008).

4. Hinton, G. E., Srivastava, N., Krizhevsky, A., Sutskever, I. & Salakhutdinov, R. R. Improving neural networks by preventing co-adaptation of feature detectors. arXiv preprint arXiv:1207.0580 (2012).

5. Sugiyama, M. & Borgwardt, K. Advances in Neural Information Processing Systems 26, 467–475 (2013).

6. Vincent, P., Larochelle, H., Lajoie, I., Bengio, Y. & Manzagol, P.-A. Stacked Denoising Autoencoders: Learning Useful Representations in a Deep Network with a Local Denoising Criterion. J. Mach. Learn. Res. 11, 3371–3408 (2010).

a

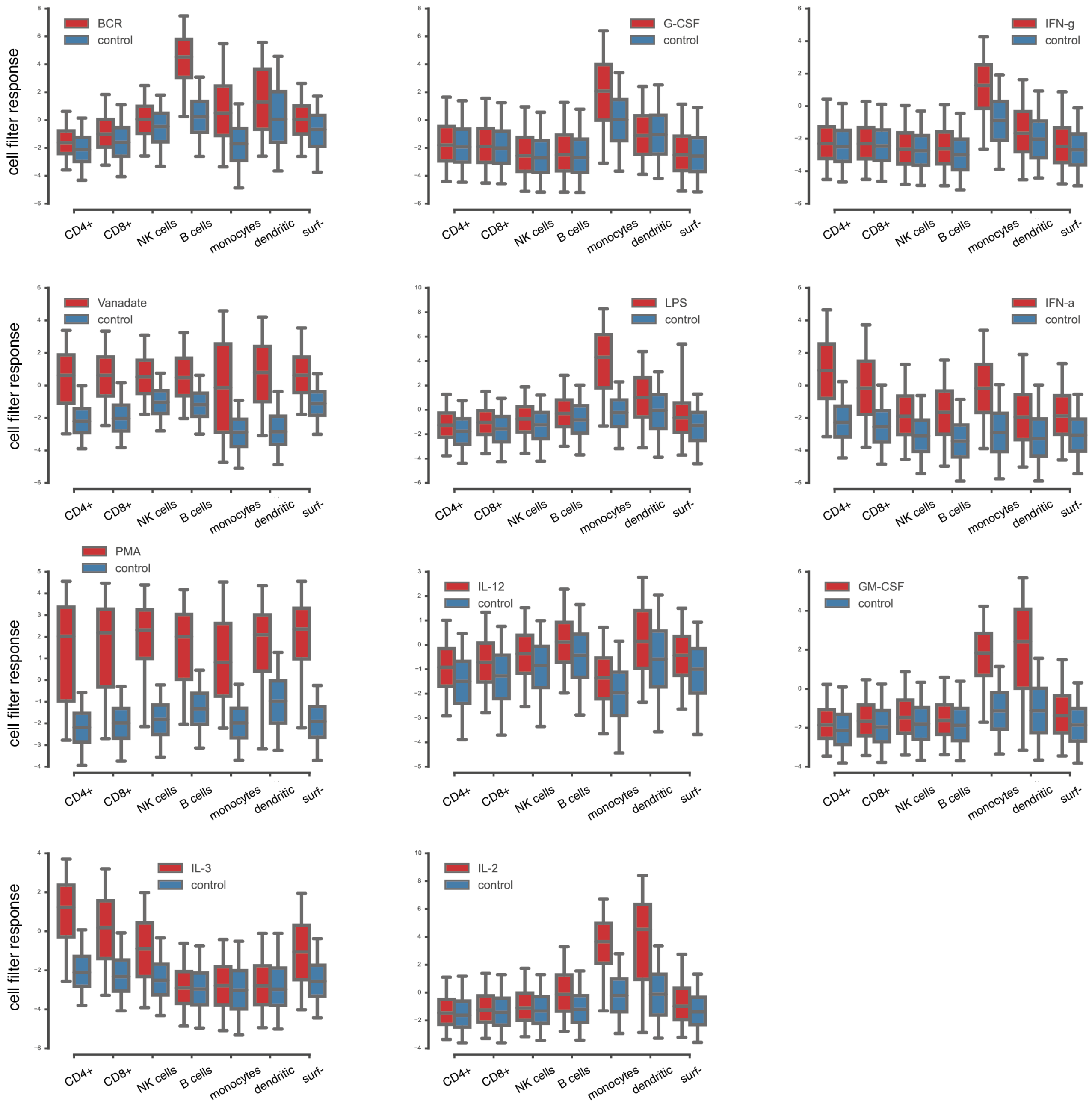
BCR
control
G-CSF
control
IFN-g
control
Vanadate
control
LPS
control
IFN-a
control
PMA
control
IL-12
control
GM-CSF
control
IL-3
control
IL-2
control
cell filter response
CD4+
CD8+
NK cells
B cells
monocytes
dendritic
surf-

b

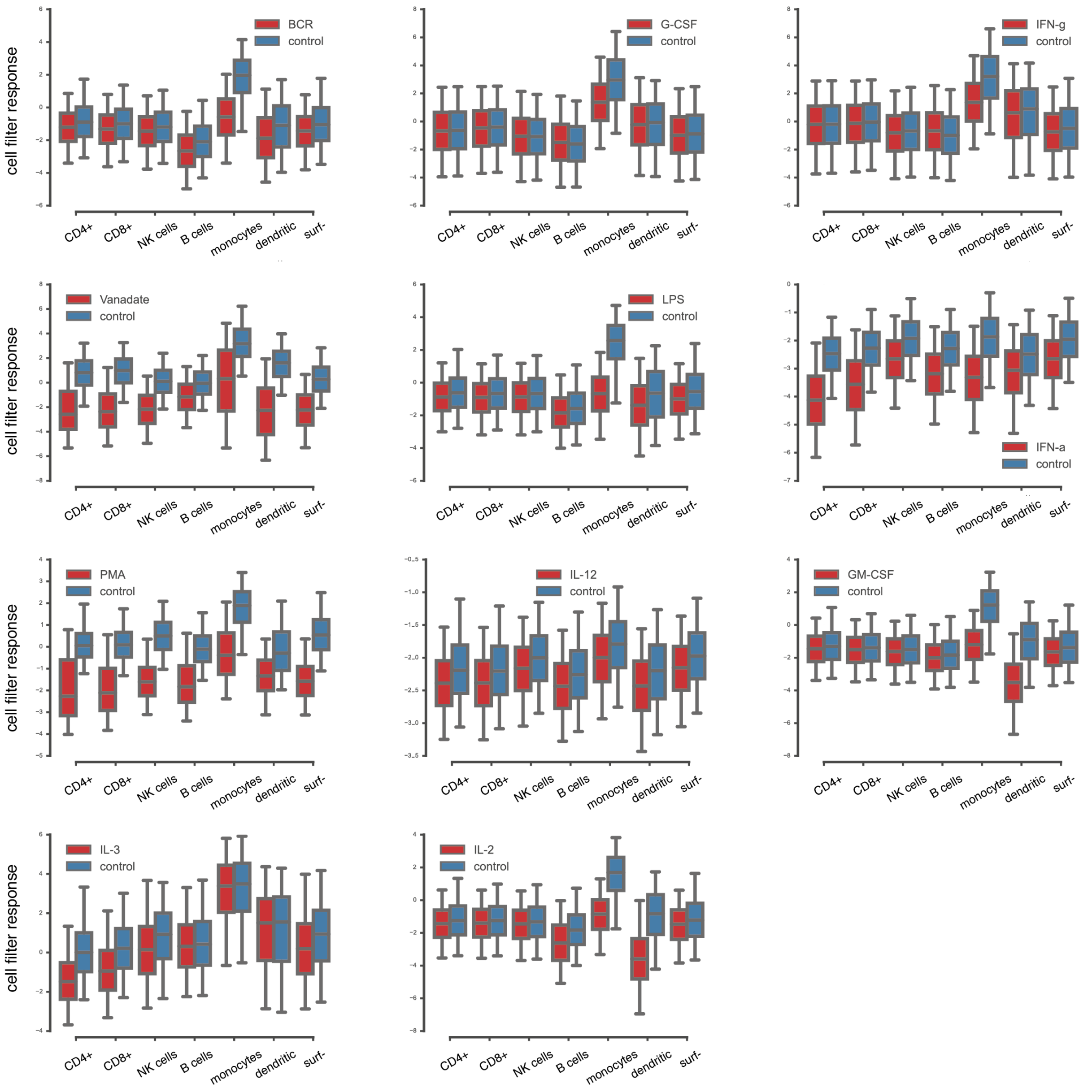

Supplementary Figure 1: Cell responses to the filter **(a)** positively associated with the stimulated condition and **(b)** negatively associated with the stimulated condition, for the paracrine agents considered in Bodenmiller *et al*. [1]. Cell filter responses are grouped by manually gated cell types.

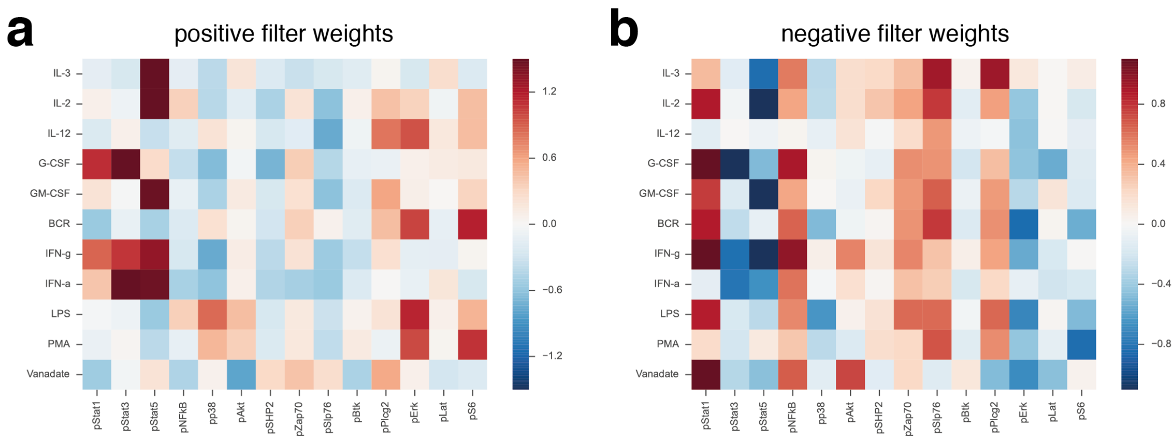

Supplementary Figure 2: Filter profiles learned by CellCnn for the paracrine agents considered in Bodenmiller *et al.* [1]. Filter profiles for filters **(a)** positively associated with the stimulated condition and **(b)** negatively associated with the stimulated condition.

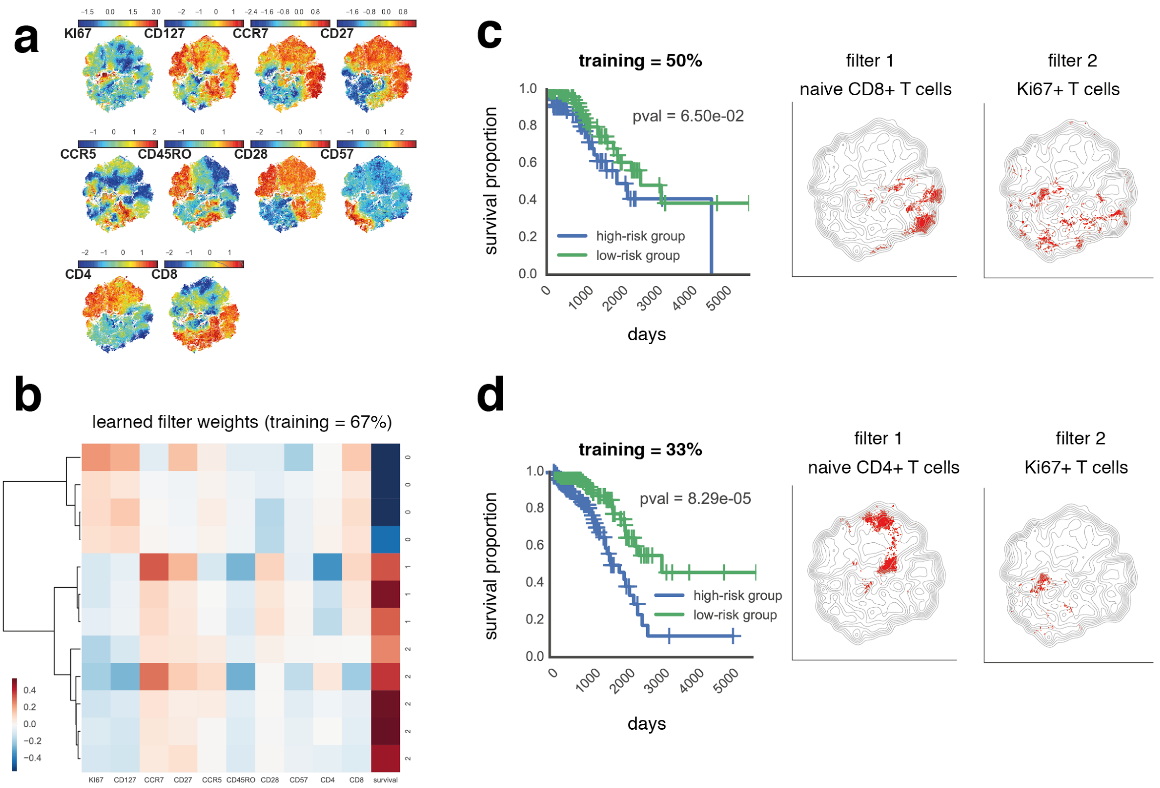

Supplementary Figure 3: Supplementary figures for the CellCnn analysis of the HIV cohort dataset. **(a)** Abundance profile of individual markers across the t-SNE projection used throughout the manuscript. The t-SNE projection was computed using samples from individuals belonging to the test cohort. **(b)** Filter weights learned by CellCnn when using 67% of the samples for training and 33% for testing. Hierarchical clustering revealed 3 distinct groups of filter weight vectors. **(c)** CellCnn analysis using 50% of the samples for training and 50% for testing. Kaplan-Meier plot for high- and low-risk patient cohort according to CellCnn survival prediction (left). Reconstruction of the cell subsets predicting AIDS-free survival in HIV-infected patients (right). Filter 1, selecting naive CD8+ T cells (similar to filter 1 in Fig. 2b), was positively associated with survival. Filter 2, selecting a Ki67+ T-cell population (similar to filter 3 in Fig. 2b), was negatively associated with survival. **(d)** Similar as (c) but using 33% of the samples for training and 67% for testing. Filter 1, selecting naive CD4+ T cells (similar to filter 2 in Fig. 2b), was positively associated with survival. Filter 2, selecting a Ki67+ T-cell population (similar to filter 3 in Fig. 2b), was negatively associated with survival.

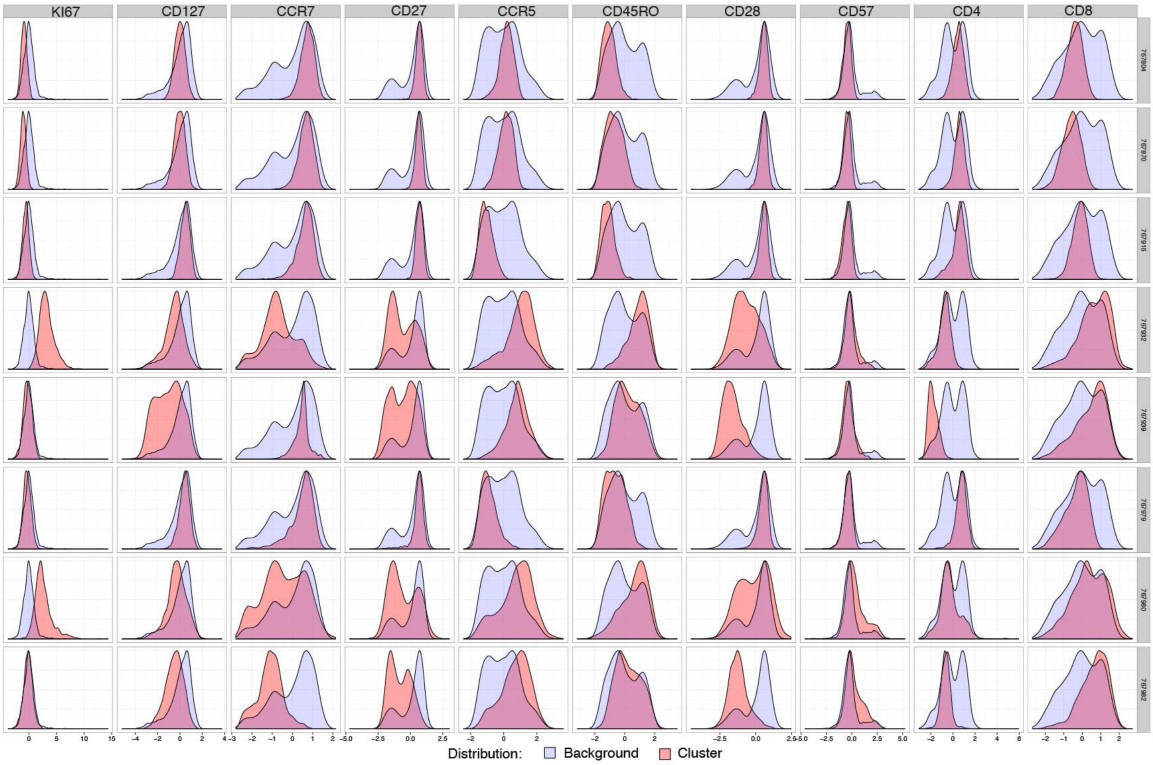

Supplementary Figure 4: Citrus analysis with publicly available code [2] of the HIV dataset on the same training/test data partition as used for CellCnn. ⅔ of the samples were used as training set and ⅓ as test set. Eight cell clusters were selected in total. From these, four clusters exhibited a CD4+ naive T cell phenotype and were positively associated with survival. The other four clusters (two Ki67+ clusters and two CCR5+ CD28- CD8+ clusters) were negatively associated with survival. The regression coefficient of Cluster 767932 (Ki67+ cluster) was one order of magnitude higher than the other coefficients denoting strong phenotype-association of this subpopulation.

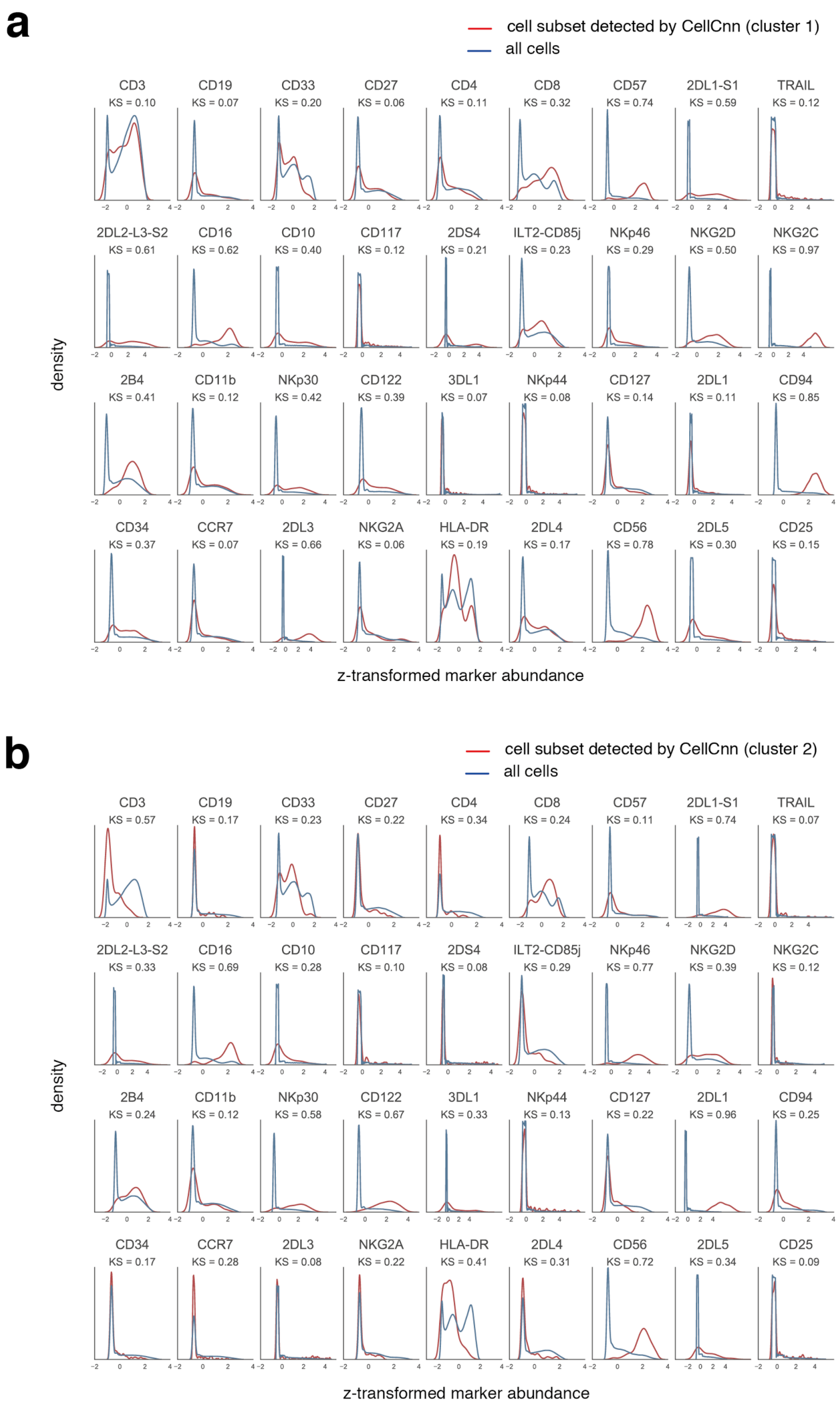

a
cell subset detected by CellCnn (cluster 1)
all cells
CD3 KS = 0.10
CD19 KS = 0.07
CD33 KS = 0.20
CD27 KS = 0.06
CD4 KS = 0.11
CD8 KS = 0.32
CD57 KS = 0.74
2DL1-S1 KS = 0.59
TRAIL KS = 0.12
2DL2-L3-S2 KS = 0.61
CD16 KS = 0.62
CD10 KS = 0.40
CD117 KS = 0.12
2DS4 KS = 0.21
ILT2-CD85j KS = 0.23
NKp46 KS = 0.29
NKG2D KS = 0.50
NKG2C KS = 0.97
2B4 KS = 0.41
CD11b KS = 0.12
NKp30 KS = 0.42
CD122 KS = 0.39
3DL1 KS = 0.07
NKp44 KS = 0.08
CD127 KS = 0.14
2DL1 KS = 0.11
CD94 KS = 0.85
CD34 KS = 0.37
CCR7 KS = 0.07
2DL3 KS = 0.66
NKG2A KS = 0.06
HLA-DR KS = 0.19
2DL4 KS = 0.17
CD56 KS = 0.78
2DL5 KS = 0.30
CD25 KS = 0.15
density
z-transformed marker abundance
b
cell subset detected by CellCnn (cluster 2)
all cells
CD3 KS = 0.57
CD19 KS = 0.17
CD33 KS = 0.23
CD27 KS = 0.22
CD4 KS = 0.34
CD8 KS = 0.24
CD57 KS = 0.11
2DL1-S1 KS = 0.74
TRAIL KS = 0.07
2DL2-L3-S2 KS = 0.33
CD16 KS = 0.69
CD10 KS = 0.28
CD117 KS = 0.10
2DS4 KS = 0.08
ILT2-CD85j KS = 0.29
NKp46 KS = 0.77
NKG2D KS = 0.39
NKG2C KS = 0.12
2B4 KS = 0.24
CD11b KS = 0.12
NKp30 KS = 0.58
CD122 KS = 0.67
3DL1 KS = 0.33
NKp44 KS = 0.13
CD127 KS = 0.22
2DL1 KS = 0.96
CD94 KS = 0.25
CD34 KS = 0.17
CCR7 KS = 0.28
2DL3 KS = 0.08
NKG2A KS = 0.22
HLA-DR KS = 0.41
2DL4 KS = 0.31
CD56 KS = 0.72
2DL5 KS = 0.34
CD25 KS = 0.09
density
z-transformed marker abundance

Supplementary Figure 5: Histograms of all measured marker abundances for the whole cell population and the cell subsets most frequently selected by CellCnn. **(a)** Cell population representative of cluster 1 in Figure 3a. **(b)** Cell population representative of cluster 2 in Figure 3a. Details on the selection of representative populations are given in **Methods**.

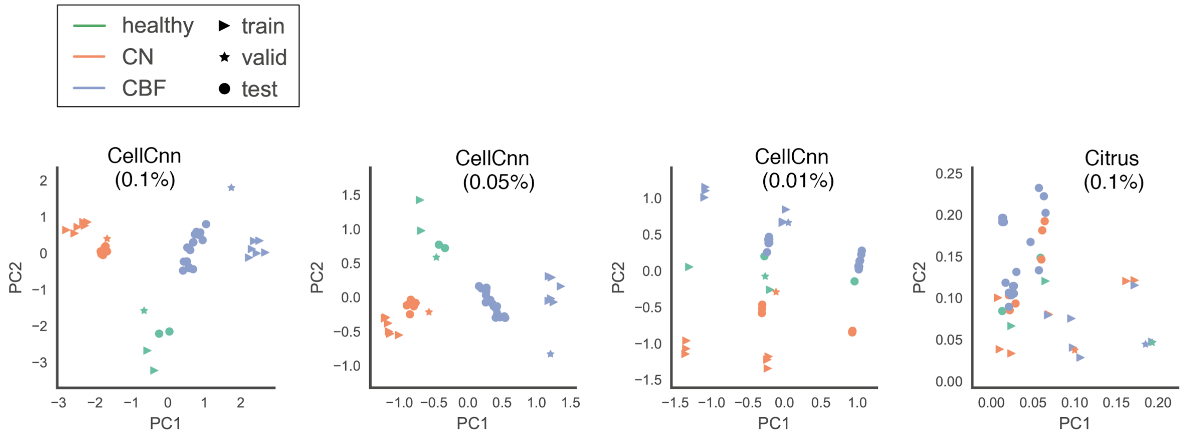

Supplementary Figure 6: CellCnn-based and Citrus-based whole-sample representations for the AML rare cell type study projected to the first two principal components. The frequency of the rare blast population considered is indicated in parenthesis.

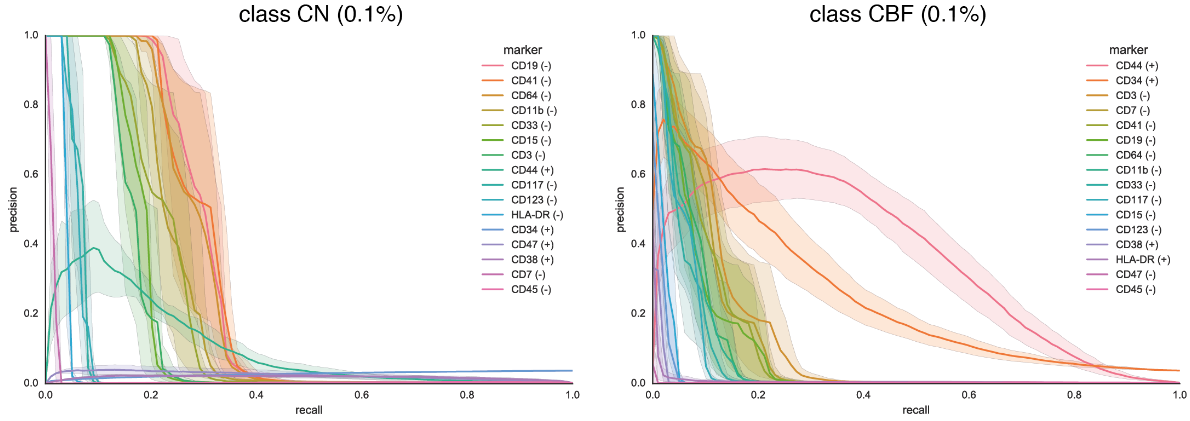

Supplementary Figure 7: Precision-recall curves on the test samples for the AML rare cell type study using single markers as predictors. The markers are ordered in decreasing order according to the area under the precision-recall curve achieved. The (+) or (-) sign indicates that overexpression/underexpression of a specific marker was used to detect the blast population.

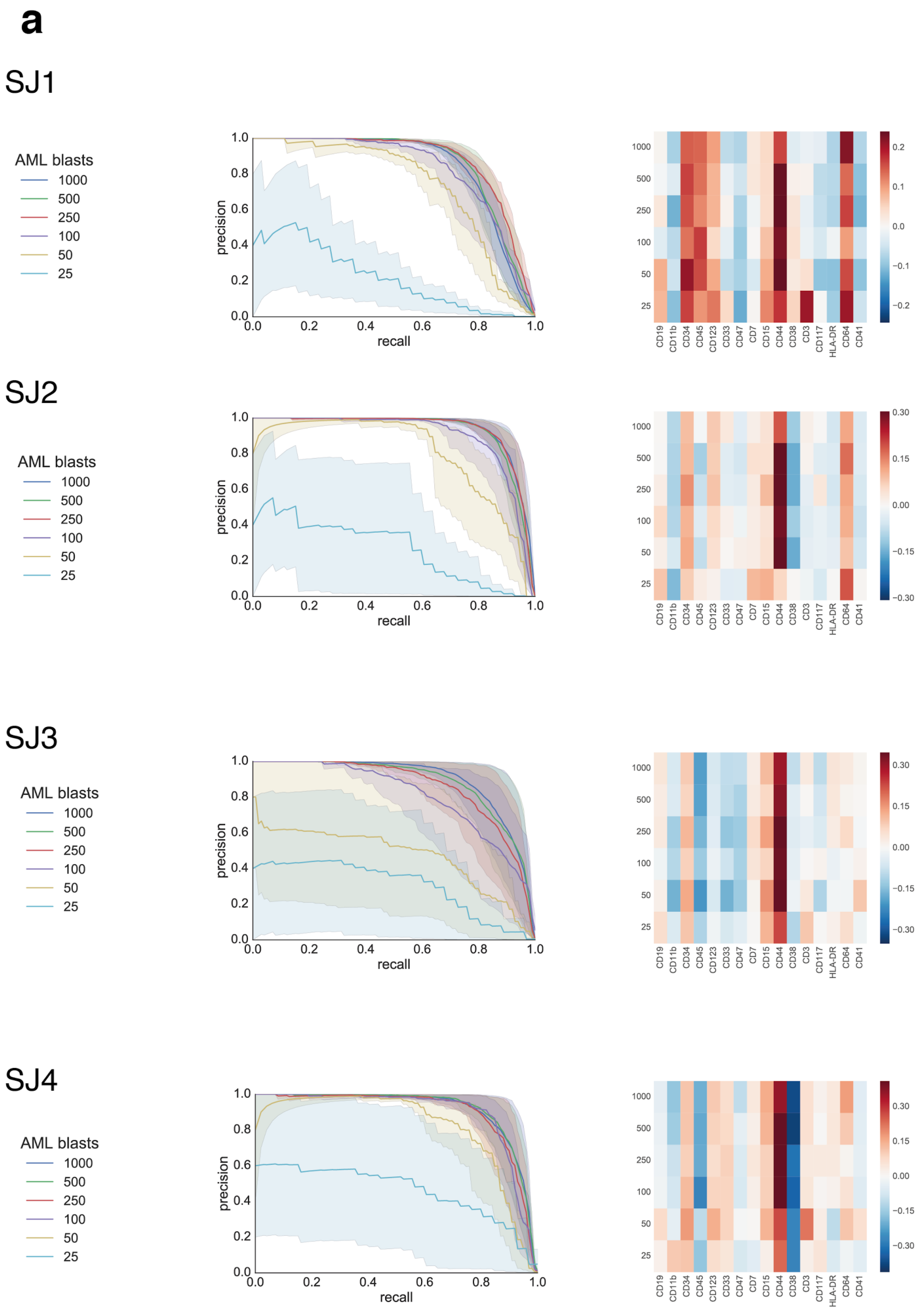
a
SJ1
SJ2
SJ3
SJ4
AML blasts
1000
500
250
100
50
25
precision
recall
CD19
CD11b
CD34
CD45
CD123
CD33
CD47
CD7
CD15
CD44
CD38
CD3
CD117
HLA-DR
CD64
CD41

SJ5

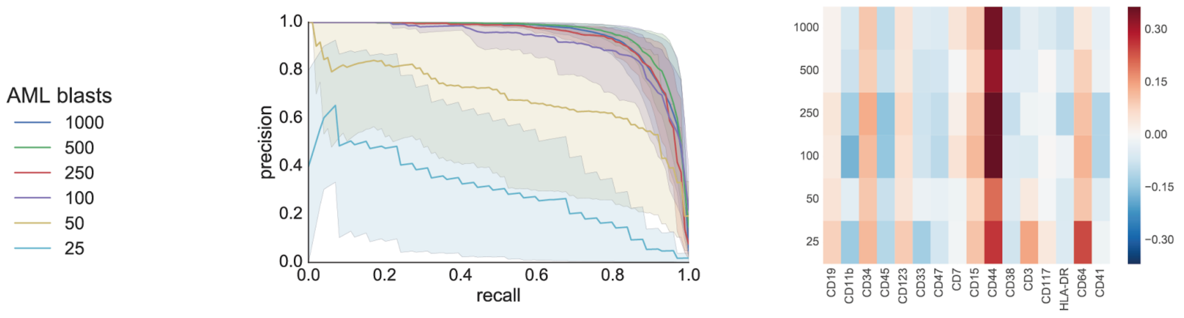

SJ7

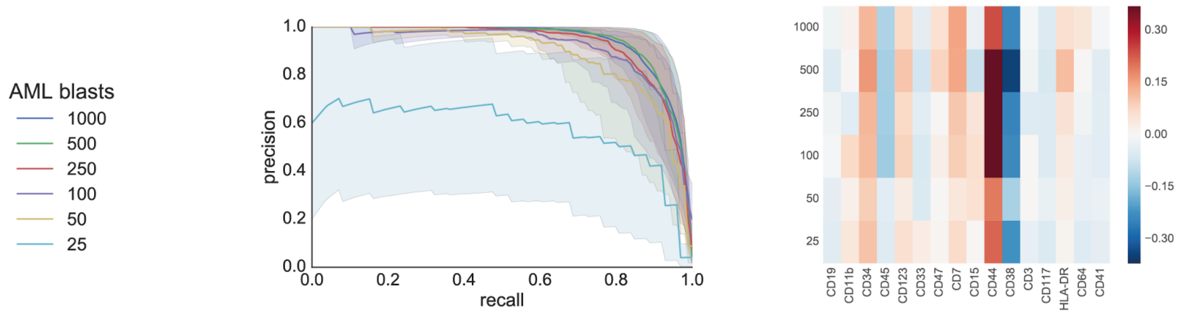

SJ10

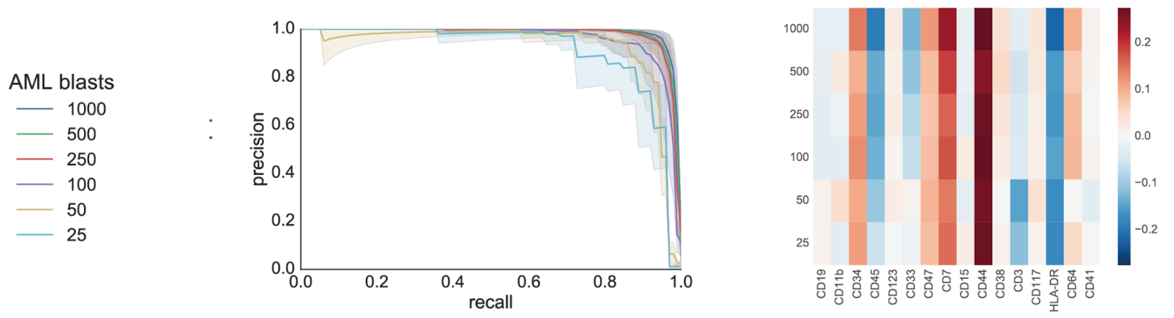

SJ12

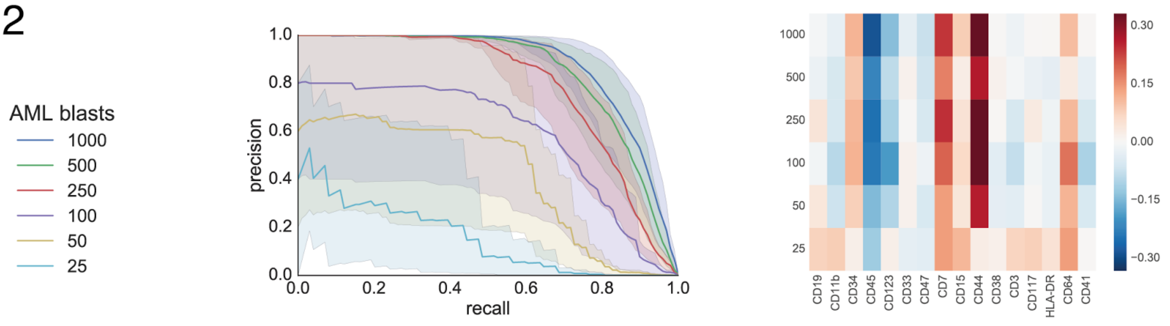

SJ13

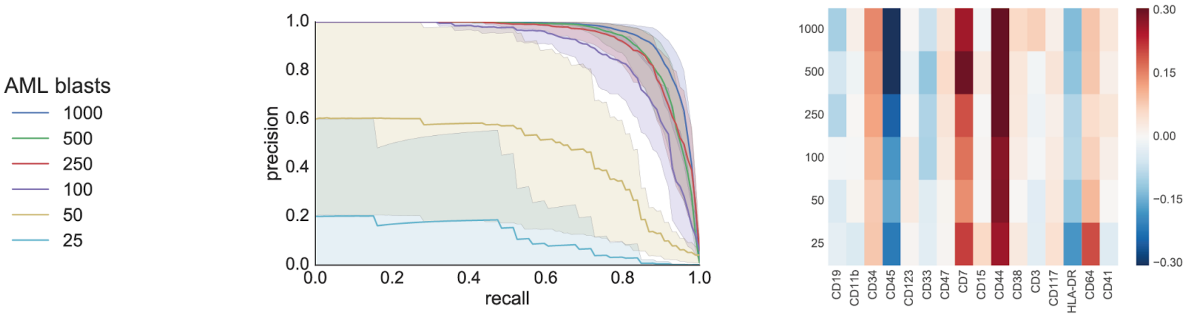

SJ14

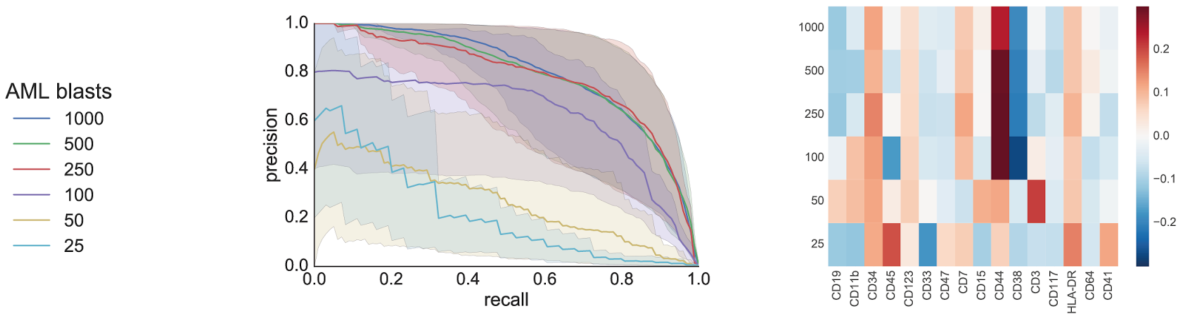

SJ15

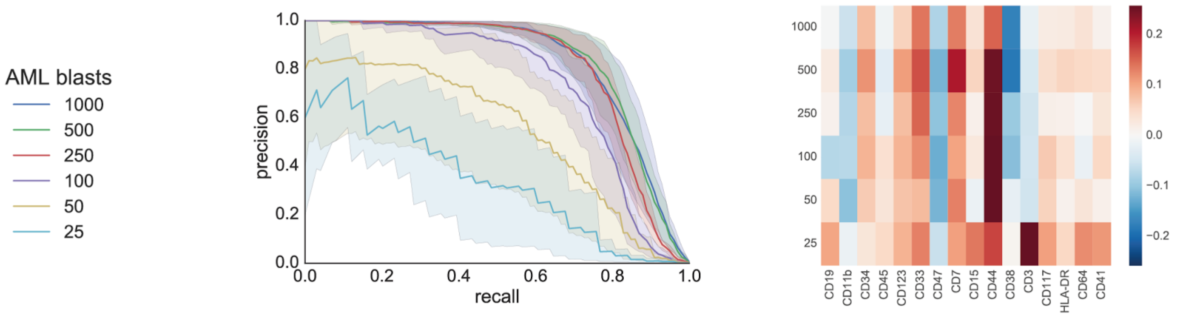

SJ16

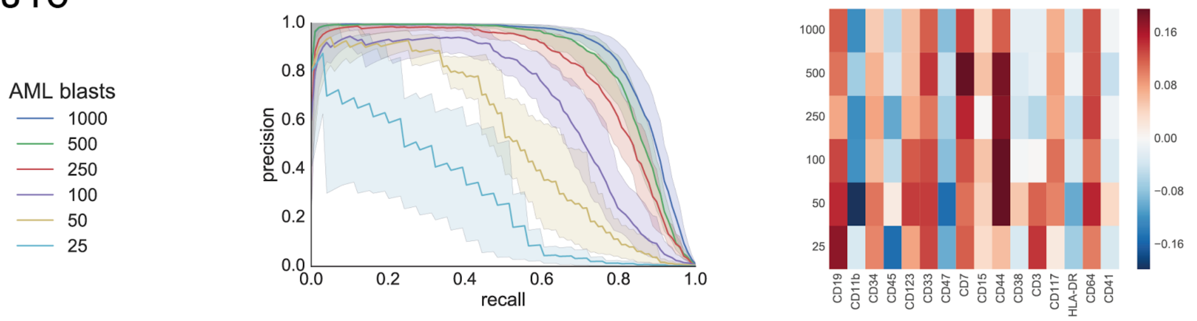

b

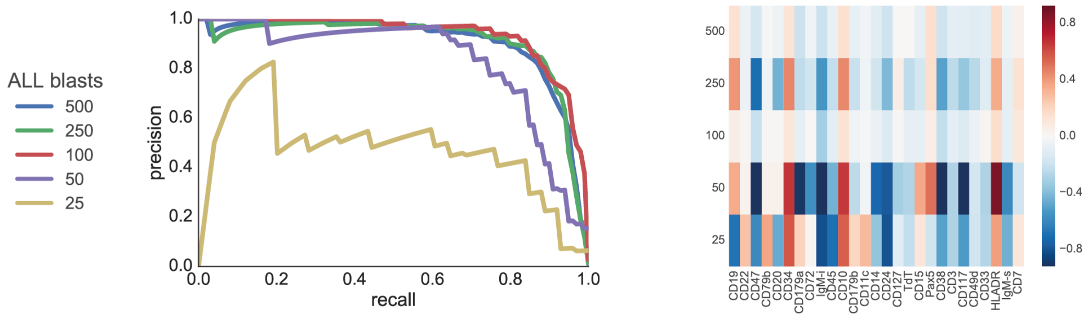

Supplementary Figure 8: Precision-recall curves and corresponding filters used to obtain the curves for the "personalized medicine" setting. Low-frequency leukemic blast populations from **(a)** different AML patients and **(b)** one ALL patient were computationally spiked into healthy bone marrow samples in order to create synthetic MRD samples. For each patient, different curves correspond to different numbers of blast cells considered. Five different healthy bone marrow samples were considered for AML and one for ALL. Therefore, average precision-recall curves are presented for AML, with shadowed areas indicating the 95% confidence interval. In the absence of validation samples, the precision-recall curves reported here were obtained using the filter that achieved the highest area under the precision recall curve (AUPRC) score among other learned filters. While overfitting cannot be assessed in this setting, our results demonstrate that, in the vast majority of cases considered, at least one filter learns to detect the rare leukemic blast population. The filter weights of the corresponding highest-scoring filters are depicted in the heatmaps.

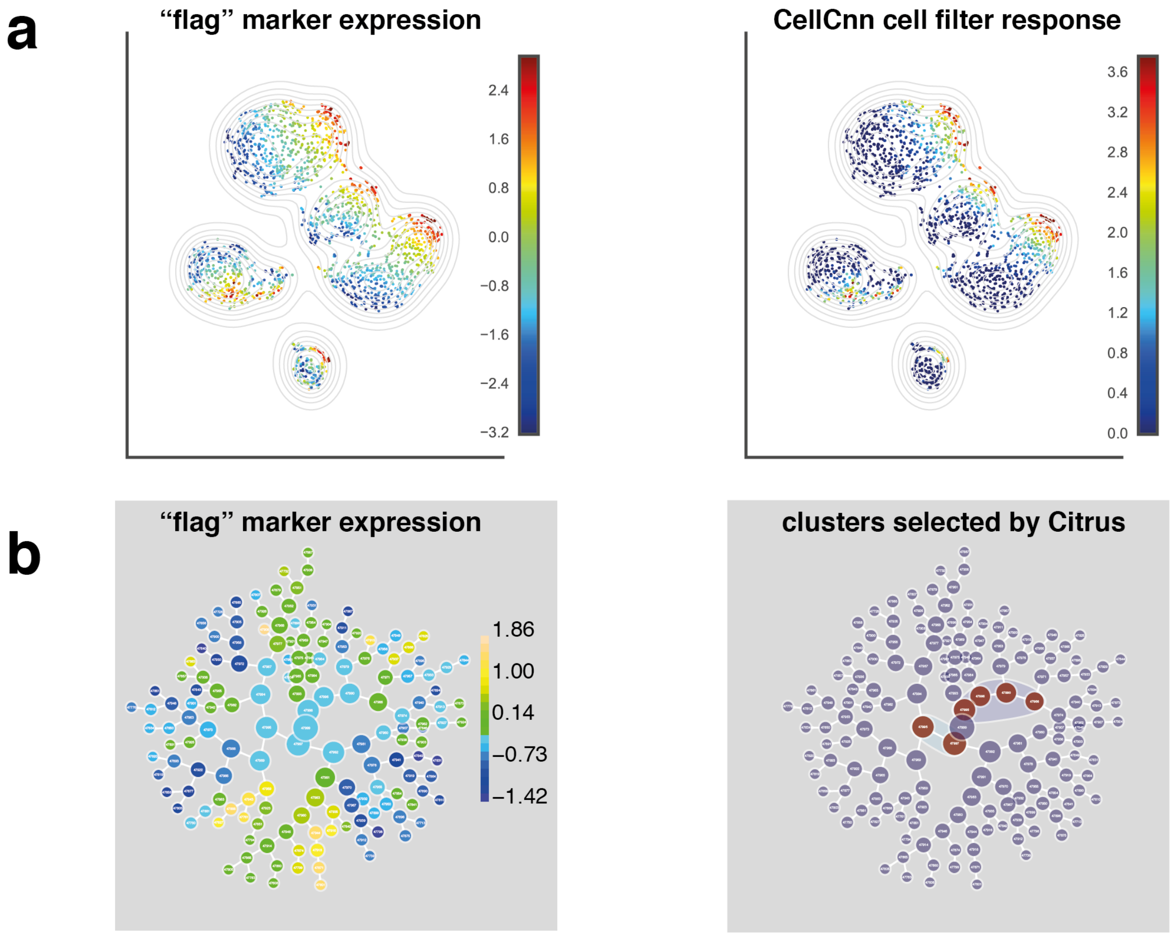

Supplementary Figure 9: CellCnn is able to detect phenotype-associated populations that do not form distinct subset clusters. A synthetic example is presented here on the basis of the PBMC dataset, only considering the ten cell surface markers measured for each cell. These markers clearly define distinct cell types. We assume two different phenotypes A and B. The only difference between these phenotypes is the activity level of one biological process that is active in a cell subset. This activity level is monitored by the abundance of an additional indicative marker (called “flag”). Samples from phenotype A only contain cells negative for the “flag” marker, generated from a normal distribution $N(\mu = -1, \sigma^2 = 1)$. Samples from phenotype B contain cells negative for the “flag” marker, but also a subpopulation of cells (frequency = 0.25%) that is positive for the “flag” marker, generated from $N(\mu = 1, \sigma^2 = 1)$. The “flag”-positive cell subset is identified by CellCnn but cannot be identified by Citrus. **(a)** The cell subset exhibiting high cell filter response coincides with the cell subset expressing the “flag” marker, although this cell subset does not form a clear cluster in terms of the measured markers. **(b)** Hierarchical representation of the clusters formed during the Citrus analysis. On the left subplot, clusters are color-coded according to median expression of the “flag” marker. On the right subplot, the clusters finally selected by Citrus are highlighted in red. The Citrus plots were produced using publicly available code [2].

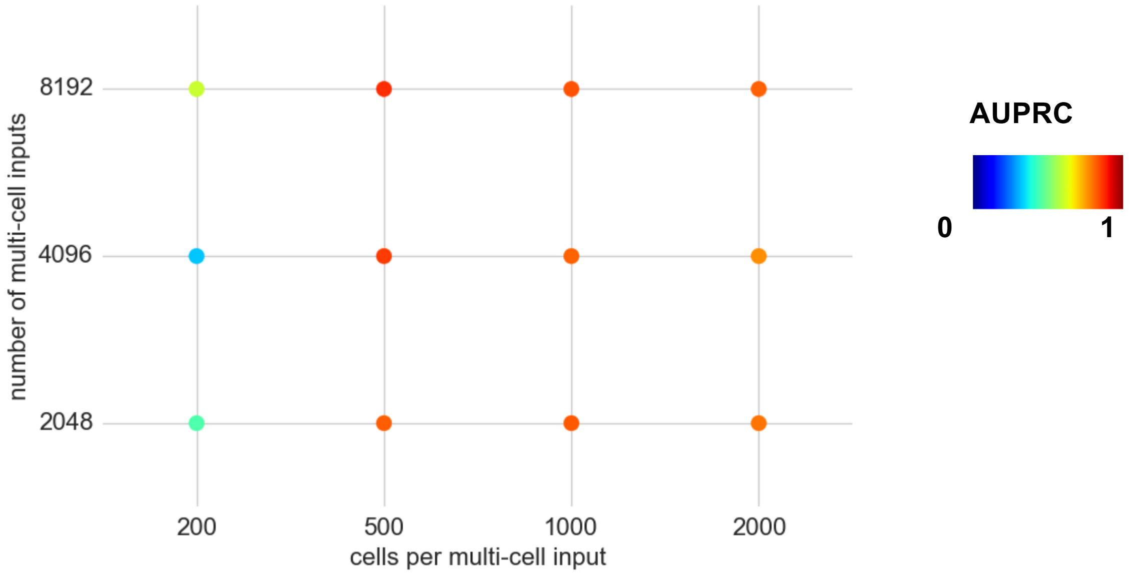

Supplementary Figure 10: CellCnn performance for different choices of multi-cell input size and number of multi-cell inputs. Grid points correspond to the different multi-cell input size/number combinations and their color-coding indicates the area under the precision-recall curve (AUPRC) of the respective CellCnn model. The average AUPRC is reported for three synthetic MRD samples, each generated by computationally spiking 300 leukemic blasts (frequency = 0.04%) from an AML patient into a healthy bone marrow sample. Each synthetic MRD sample was then compared with a pool of healthy cells from four different healthy bone marrow samples.

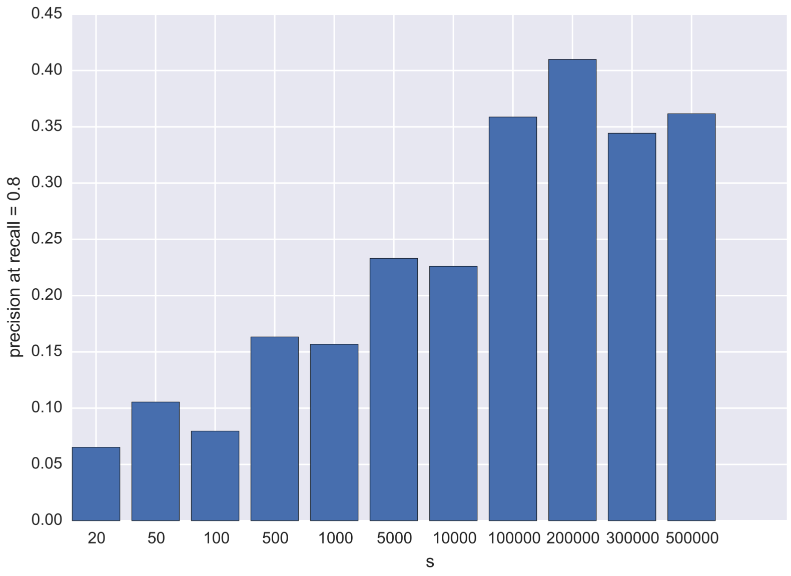

Supplementary Figure 11: Evaluation of the outlier detection algorithm on a synthetic AML MRD-like sample (AML blast frequency = 0.2%) for different values of the parameter $s = |S|$, where S is a set of observations randomly sampled from the reference class. The outlierness score for individual test observations is defined as their distance to their closest neighbor in the set S. We finally used s = 200,000 for comparing with CellCnn.

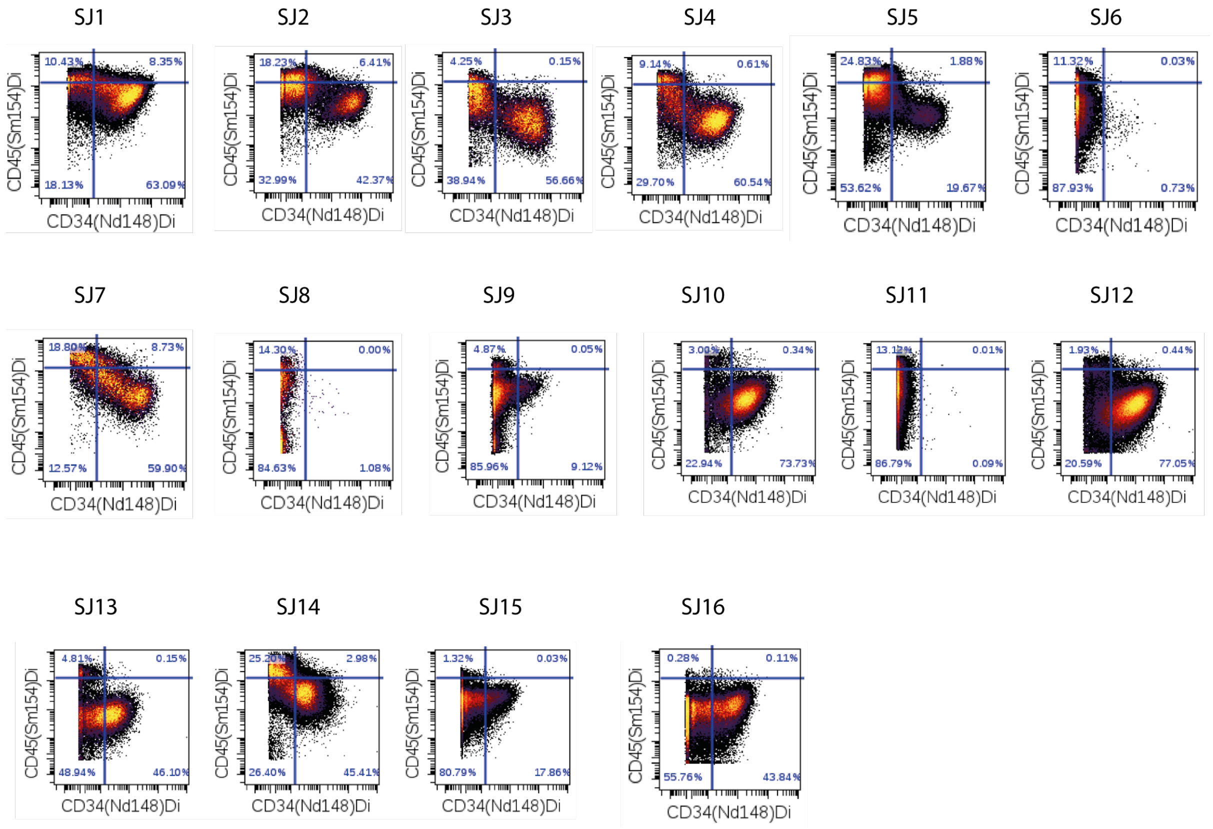

Supplementary Figure 12: Gating scheme for isolating CD34+ CD45mid blast populations in different AML patients. We have further analyzed samples from patients with total blast frequency > 10%.
